# Isotype diversity of antibodies specific for component allergens in the context of allergic diseases: eosinophilic esophagitis (EoE), asthma, and the alpha-gal syndrome (AGS)

**DOI:** 10.3389/falgy.2024.1488857

**Published:** 2025-02-04

**Authors:** Thomas A. E. Platts-Mills, Matthew H. MacCallum, Jeffrey M. Wilson, Lisa J. Workman, Elizabeth A. Erwin

**Affiliations:** ^1^ Division of Allergy & Clinical Immunology, Department of Medicine, University of Virginia, Charlottesville, VA, United States; ^2^ Department of Allergy at Nationwide Children’s Hospital, Ohio State University College of Medicine, Columbus, OH, United States

**Keywords:** isotype of allergen specific antibodies, eosinophilic esophagitis, the alpha-Gal syndrome, IgG4 specific for wheat and milk, IgE to alpha-gal

## Abstract

From the earliest days of studying the reagins in allergic sera that give rise to the Prausnitz-Kuestner reaction, there was evidence that there were other types of antibodies (Ab) specific for allergens, particularly those induced by immunotherapy. By 1980, not only was IgE recognized and could be measured, but the presence of other isotypes including IgG and IgA in patients with IgE was well established. From that time onwards the development of monoclonal antibodies made it possible to distinguish and measure antibodies of other isotypes such as IgG4, IgG2, and IgG3. Over the past 40 years two things have dominated the field- firstly, the techniques for measuring isotype specific antibodies to allergens have improved steadily. Secondly, several different allergic diseases or phenomena have been identified in which isotype diversity of the antibodies has become a major issue. Prior to 1990 only occasional cases of eosinophilic esophagitis (EoE) had been identified, but since then they have become common. Most of the cases have positive skin tests and/or IgE Ab to cow's milk or wheat, but it became obvious that most cases of EoE are not primarily related to IgE. Today it is clear that IgG4 Ab to these allergens play a significant role in cases of EoE. In 2000 the first reports of children developing tolerance to cat allergen appeared. Today it is clear that this tolerance depends on high levels of IgG4 antibodies and there is increasing evidence that the IgG4 response is primarily against *Fel d 1*. The most recent novel allergic disease is the alpha-gal syndrome (AGS). This condition is based on IgE antibodies specific for the oligosaccharide galactose alpha,1-3-galactose, which are primarily induced by tick bites. However, in this case it was already well known that all immunocompetent primates have made IgG and IgM antibodies to this oligosaccharide. Furthermore, it is not clear whether the IgG isotypes, particularly IgG1 and IgG3, play a role in the inflammatory response to the oligosaccharide. Overall, it is clear that current and future investigation of allergic diseases requires careful assessment of allergen specific antibodies of diverse isotypes in addition to IgE.

## 1 Introduction

Although there were techniques for identification of isotype specific antibodies prior to the discovery of IgE, they were not simple to carry out and could not provide quantitative results. These included radio-immune electrophoresis (RIE) which never came close to being suitable for use in studying large cohorts or clinical use.

There were three major issues in relation to studying the isotypes of allergen specific antibodies:

I. Was the objective to measure antibodies specific for a source of allergens such as pollen or cat extract, or to evaluate antibodies specific for individual proteins or components from each source?
II. Defining the isotypes requires either a polyclonal antibody where the specificity can be trusted or a monoclonal antibody that can be used to define the specific antibodies, of that isotype.
III. Finally any assay requires a technique to immobilize the isotype specific antibodies binding to allergen specific antibodies of that isotype. Good examples are precipitates of the isotype specific antibodies with the labeled allergen which was used; or a solid phase to which the allergen or allergens are bound. This allows attachment of antibodies from a patient's serum so that a labelled isotype specific antibody can be used to identify and quantify the allergen specific antibodies.

**Figure 1:**
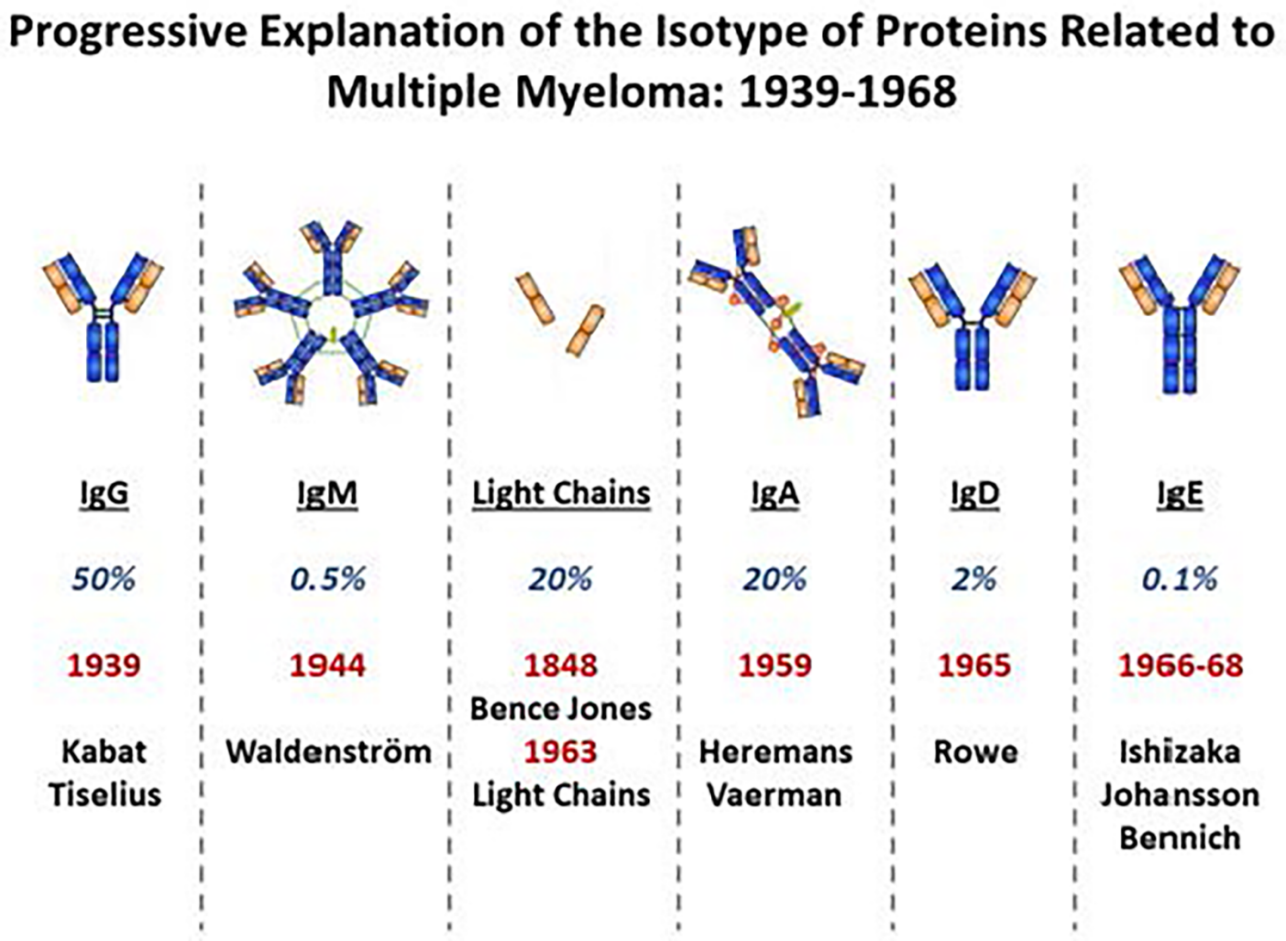
Progressive explanation of the isotype of proteins related to multiple myeloma: 1939–1968.

The availability of polyclonal antibodies against different immunoglobulin isotypes in general followed the identification of the isotypes which included finding relevant myeloma proteins (1–4) (Figure 1). In 1965 the biggest challenge was to identify the “reaginic” antibodies that were capable of transferring allergen sensitization from an allergic patient to the skin of a non-allergic patient (5–7). This was because there appeared to be and, indeed was, very little antibody of this kind in the circulation; and the isotype of almost all of the individual myeloma proteins that had been studied was found to be one of the recognized isotypes (Figure 1). The full identification of IgE came from a successful collaboration between two groups who were using completely different approaches. Dr. Kimishige Ishizaka first recognized the importance of the question while working with Dr. Dan Campbell at Cal Tec, he then moved to Denver to purify the antibodies that could give a positive Prausnitz-Kustner (P-K) reaction, using serum from a patient who was highly allergic to ragweed (5, 8). At the same time, Dr. Gunnar Johansson identified a myeloma patient where the monoclonal protein present in his serum could not be classified (7). He and Dr. Hans Bennich in Sweden purified the myeloma protein, and then working with Dr. Stanworth and Dr. John Humphrey in the UK, demonstrated that this protein could block the P-K reaction (9). At that point the two groups collaborated and proved that they were studying the same protein, which Ishizaka had already named IgE (8, 10). Within one year after this, ie in 1967, the Swedish group had established the Radio allergosorbent-test (RAST) which by 1970 was available for measuring IgE to multiple different allergens and became widely used to diagnose allergic disease (11).

For most of the isotypes such as IgA, IgM, and IgG it was relatively easy to purify the protein and myeloma proteins were available. However, making truly specific antibodies to the different forms of IgG such as IgG2, IgG3, and IgG4 was difficult. So the major developments over the next 20 years included (i): investigating different forms of solid phases to absorb allergens, (ii) purification of multiple specific allergens, and (iii), the development of monoclonal antibodies first to IgE and subsequently monoclonals to IgA, IgM, IgD as well as those to IgG4, IgG3, and IgG2. A major problem with the assays for other isotypes is that they had then and still have much higher background binding to the solid phase compared to IgE and because of this have to be diluted at least 50 fold in order to get reliable assays.

## 2 Precipitation technique for measuring IgG and IgA antibodies to radiolabeled antigens

The first known precipitation assay was developed by Dr. Farr in Denver, using radiolabeled insulin incubated with the serum to be tested, following which the IgG was precipitated by adding 33% saturated ammonium sulphate. The precipitate could then be washed with ammonium sulphate and counted via gamma counter.

The next approach used radiolabeled allergens and could not be applied to extracts of allergens because there is no way to radiolabel an extract such that the different proteins are consistently labeled. The purified allergen was incubated with the serum samples and then the relevant isotype of immunoglobulin was precipitated with an isotype specific polyclonal antibody. Interestingly, the best-known allergens that had been purified in the early 1970s were Ragweed Antigen E (now Amb a 1) and rye grass antigen Rye 1 (Lol p 1). Radiolabeling started with I^131^ but by 1970 was using the Chloramine T technique and I^125^ which was much safer (12). In 1976 the Hopkins group, led by Larry Lichtenstein, Phil Norman, and Kimi Ishizaka, and also including David Marsh who initially purified Lol p 1, published a paper on IgG and IgA antibodies to Antigen E in nasal washings (13). That paper established that antibodies of the non-IgE isotypes were present in nasal secretions of patients with ragweed hay fever but were not present in secretions from non-allergic subjects (13). Those results together with subsequent studies on isotype specific antibodies in the sera of allergic patients demolished the then prevalent view that allergy might be a form of immunodeficiency and equally established that the immune response containing IgE consistently included these other isotypes (14, 15).

At that point, in 1974, the senior author of this review moved to work for the Medical Research Council in London. He also transferred several critical reagents including 5 mg of purified Lol p 1, a gift from Dr. Marsh, which was the basis of a PhD thesis further defining the local immune response to pollen allergens (12, 14, 15). In addition, he brought with him a unit of plasma from the second IgE myeloma patient, P.S., who had by then been identified, in the USA, by Kimi Ishizaka.

## 3 Purification of allergens from the major indoor sources

Purification of allergens from the indoor sources that had been recognized as “causes” of asthma was essential for further study of the immune response to these allergens, but also made it possible to develop assays to measure the quantities of allergen in houses (16). The first of the indoor allergens to be purified was Cat-1 from *Felis domesticus* (now Fel d 1) by Jack Ohman in 1974 (17) which was followed by the purification of mite allergen F_4_P_1_ (now Der p 1) in 1978 (18). Notably the first purified allergens from grass, ragweed, cat, and dust mite were all purified before cloning of allergens or monoclonal antibodies had been developed. Using classical immunochemistry, as defined by Dan Campbell or, Kabat & Mayer, purification of an allergen was likely to identify an allergen that was present both in the extract used for purification and in the environment, in a significant physical quantity (17–19). By contrast using cloning to identify allergens it is possible to identify proteins that are a significant allergen but are not physically present in a large quantity (www.allergen.org). The physical abundance of allergens may be an important factor in understanding the relative quantities of IgG4 and IgE antibodies induced by allergens that are related to a given source (20).

Measuring specific IgG4 antibodies to allergens is difficult because it is not easy to make fully specific polyclonal antibodies to this isotype while monoclonal antibodies do not make precipitates. The technique we used between 1984 and 2000 involved incubating the serum with a radiolabeled antigen, then adding a mouse monoclonal antibody to IgG4, and finally precipitating the mouse monoclonal antibody, together with the specific antibodies and labelled antigen, using a polyclonal antibody to the mouse IgG. The assay depends on having polyclonal antiserum that is specific for mouse IgG and has no binding to human IgG. However, most polyclonal antibodies to mouse IgG have significant cross reactivity with human IgG and had to be absorbed repeatedly over a human IgG column. After that absorption they tend to be rather poor at precipitation (21). None the less we carried out multiple studies with this technique including the study on middle school children that showed that high level exposure to cat allergens can produce high levels of IgG4 to Fel d 1 with only moderate levels of sIgE (20). That study provided the first evidence that the tolerance that can occur among children living in a house with a cat is related to the increased production of both IgG and in particular IgG4 specific to Fel d 1 (22). The possibility that an IgG4 based monoclonal antibody to Fel d 1 would be used therapeutically for cat allergy was recently studied by Regeneron using an antibody developed by Dr. Orengo (23).

## 4 ELISA assays for sIgE and other isotypes of antibodies to allergens

Enzyme Linked Immunosorbent Assays (ELISA) generally use a plate with multiple wells where the antigen or allergen has a relatively small surface area to bind to. Assays of the ELISA form have a rather poor reputation for measuring specific IgE antibodies primarily because many sera from allergic patients also have IgG antibodies which can easily block binding of IgE antibodies to the small quantities of allergen on a plate. On the other hand, ELISA assays may be effective with other isotypes. A recent example comes with assays of antibodies specific for galactose-1,3-galactose. Two different groups have used ELISA assays to measure specific IgG and IgA antibodies as well as the range of IgG isotypes – such as IgG2, IgG3, and IgG4 (24, 25). The results provided evidence that IgG4 antibodies to alpha-gal are either absent from or present in very low qualities in sera in patients with the alpha-gal syndrome (AGS), when they first present.

## 5 Modern developments in allergen specific assays for antibodies of several different isotypes; but specifically IgE and IgG4: ImmunoCAP and ImmunoCAP ISAC

Progressive developments of the RAST approach have led to major changes in the solid phase leading up to a high-capacity sponge, and an automated technique for washing this sponge between the phases of the assay. Initially the Phadia UniCAP technique and subsequently the ImmunoCAP technique requires a significant amount of serum for each assay (20–40 μL per test). This volume severely restricts the number of assays that it is possible to carry out. However, the high capacity of the sponge makes it possible to assay sera for IgE with little risk of inhibition by IgG antibodies to the same allergen. The really important feature of the modern automated technique is that the method for binding the allergen and washing the solid phase makes it possible to get results in three hours with low background on the assays. In turn, this makes it possible to provide reliable assays of quantities as low as 0.1 IU/mL of IgE. Values this low are, in most cases, of little relevance clinically and many investigators use a level of detection (LOD) of 0.35 IU/mL. Today well over 100 allergens are available commercially on ImmunoCAP either as allergen extracts or specific allergen proteins in a natural or recombinant form usually referred to as components.

As part of the development of the ImmunoCAP the company manufactured a form of the sponge heavily coated with streptavidin, which was not initially available for commercial use or research. In 2005 we collaborated with Phadia and developed the method for binding biotinylated Fel d 1 or Der p 1, to the streptavidin CAP (26). That technique played a major role in the investigation of anaphylactic reactions to the monoclonal antibody Cetuximab and also in the early investigation of the alpha-gal syndrome (27, 28).

**Figure 2:**
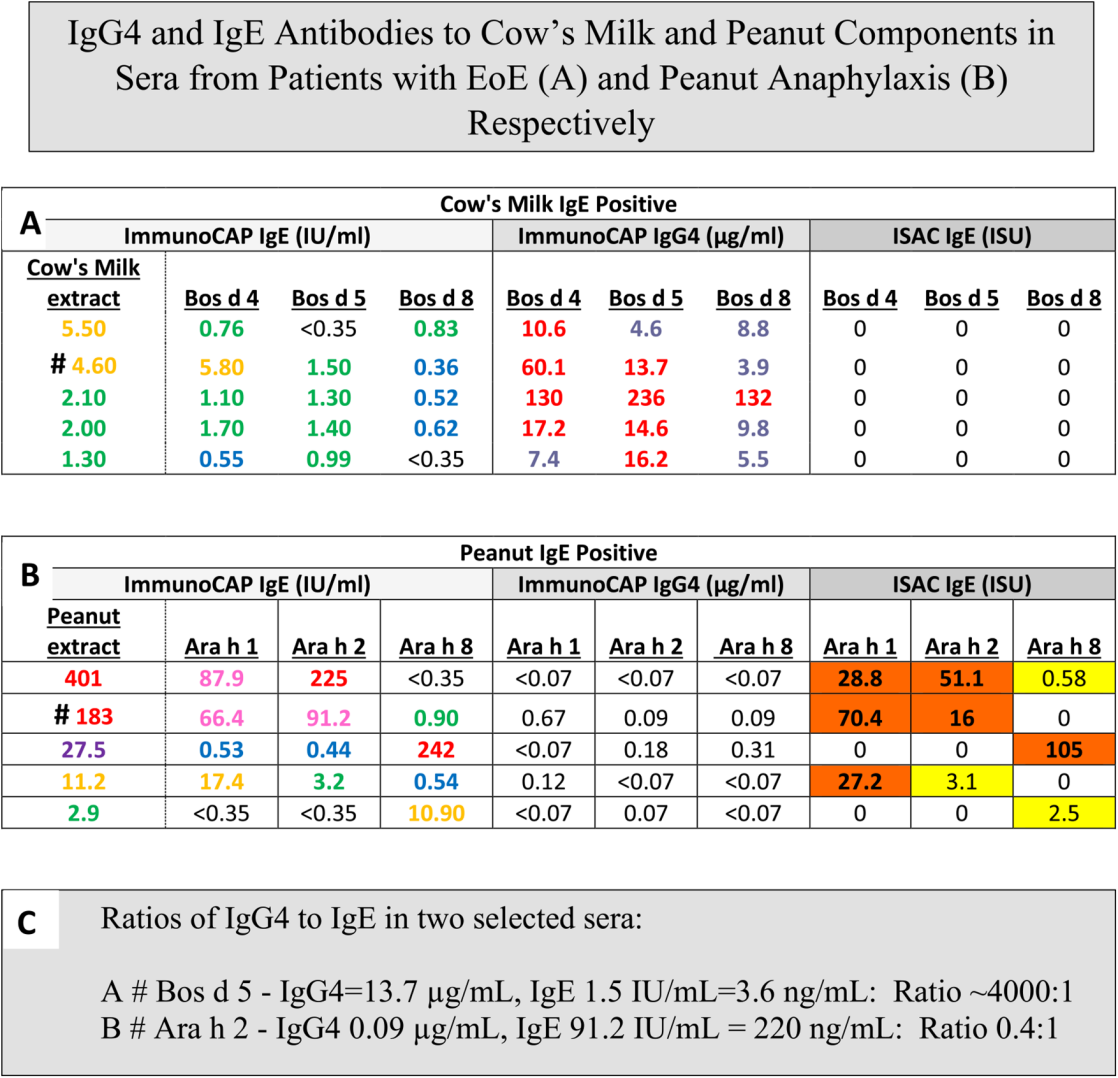
IgG4 and IgE antibodies to cows milk and peanut components in sera from patients with EoE **(A)** and peanut anaphylaxis **(B)** respectively. Values of sIgE measured either with ImmunoCAP or ISAC compared to sIgG4 measured with ImmunoCAP Only: ImmunoCAP results are given as IgE in IU/mL, IgG4 in ug/mL. ISAC results are given as ISAC Standardized Units (ISU). Values are shown for children age 6–8 with Eosinophilic Esophagitis **(A)**, reports of recent peanut anaphylaxis **(B)**, or a presentation of the ImmunoCAP *p* values converted to ng/mL to allow calculation of ratios of sIgG4:sIgE **(C)**.

ImmunoCAP ISAC (ISAC) is confusingly named because the term CAP is meant to relate to the high capacity solid phase on the sponge while ISAC is the opposite of high capacity. The chip used in ISAC has very small drops of each allergen extract placed in triplicate. The binding of the IgE or IgG4 in patient serum is then followed by the appropriate fluorescent labeled monoclonal antibody (29). The actual quantity of allergen on each spot is approximately 1 ng, and needless to say it is easy for elevated levels of sIgG4 or sIgG to block binding of sIgE to the same allergen. An excellent illustration of this effect came from a study designed to use ISAC to predict the appropriate diet for treating Eosinophilic Esophagitis (EoE). In that study, only one of the first 15 patients enrolled had sIgE to milk as judged by ISAC, presumably because patients with EoE have high titer to IgG4 to milk proteins (30, 31). Treatment with a diet that did not avoid milk or milk products produced no benefit clinically or histologically and the study had to be discontinued (30). On the other hand, using ImmunoCAP a large proportion of the patients with EoE have detectable, but low sIgE to milk and many of those patients respond well to a diet that simply avoids milk (32, 33) (Figure 2).

## 6 Use of isotype specific assays for antibodies to allergen extracts or purified proteins in the investigation of Eosinophilic esophagitis (EoE)

**Figure 3:**
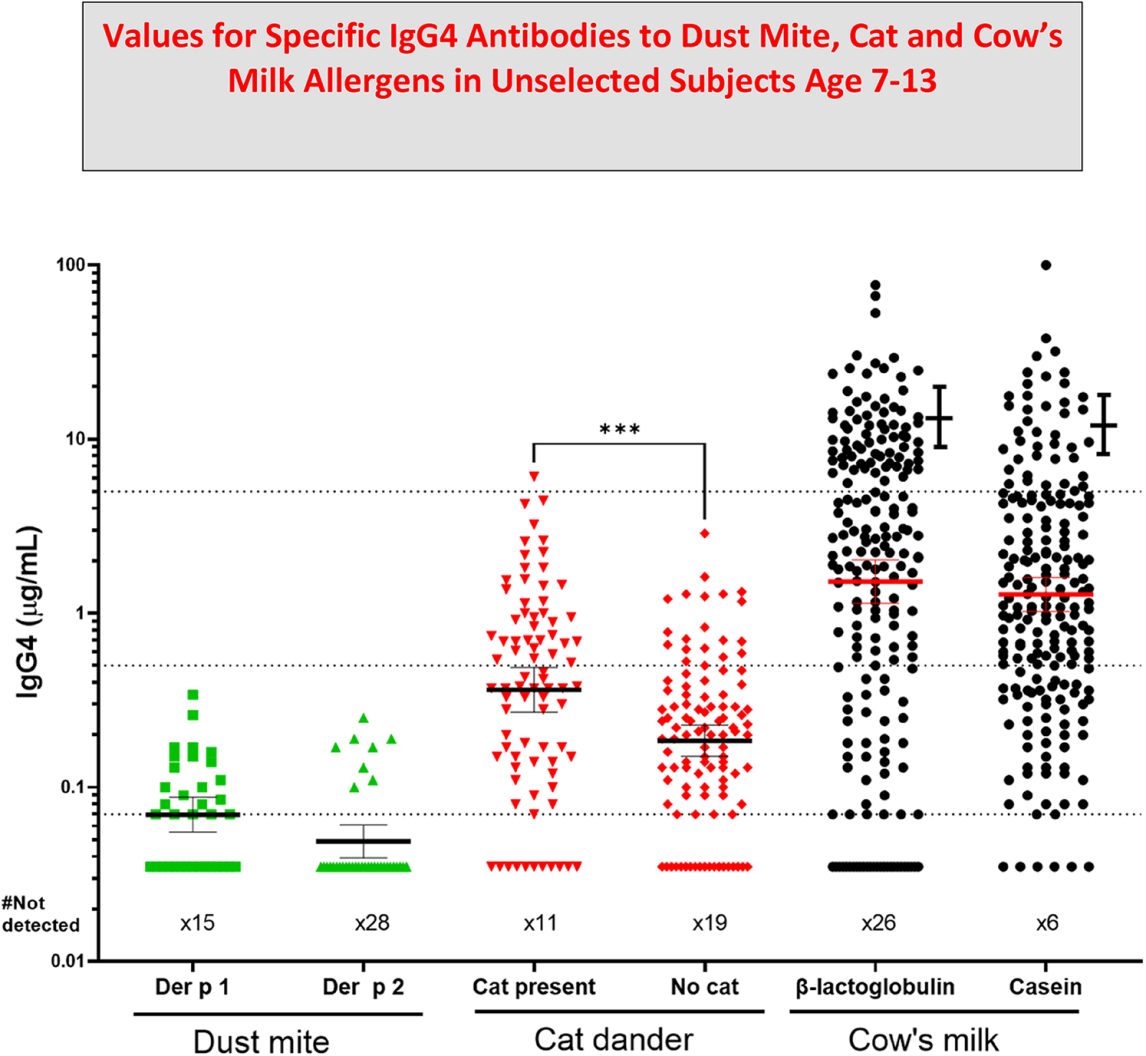
Values for specific IgG4 antibodies to dust Mite, Cat and Cow's Milk allergens in unselected subjects age 7–13. Allergen specific IgG4 (sIgG4) levels in sera from teenage participants in birth cohorts includes data from three published studies (20, 22, 30, 36). Error bars next to the data for IgG4 to cow's milk are values for age similar cases of Eosinophilic esophagitis (EoE) (31). Values for IgG4 to cat dander were compared using Mann-Whitney U test; (*p* values less than 0.001). GM Mean values are shown with 95% confidence intervals.

The first cases of EoE were recognized as early as the 1970s, however the symptoms were not widely recognized until 1990. At that time there was already evidence that most of these patients would respond to diet. The response to an amino acid based diet was almost 100%, while a six food diet was 65%–80% effective and a diet based simply on avoiding milk and dairy products could produce a major improvement in almost 50% of the cases (32, 33). Initial attempts using a diet based on skin tests, serum IgE assays or patch tests were not impressive (34). However, using ImmunoCAP to assay serum sIgE to milk or wheat it was clear that a large proportion of these patients had made a measurable IgE response to those two foods that were known to be clinically the most relevant to symptoms and esophageal inflammation. In 2014, the gastroenterology and immunology groups in Salt Lake City led by Kathie Peterson together with Fred Clayton and Gerry Gleich reported dramatic findings relating to the relevance of IgG4 to EoE (35). They actually started that study as a trial of Omalizumab (anti-IgE) treatment in patients with EoE and those results were reported as negative. However, during the study they stained the biopsies from patients using anti-sera to IgE, IgG, and IgG4. The results demonstrated that there were striking intercellular deposits of IgG4 in esophageal biopsies from patients with EoE. Given that IgG4 antibodies are fully soluble and only have a molecular weight of 160,000 it seemed unlikely they were accumulating in the wall of the esophagus without some form of cross linking in relation to the relevant allergen (35). Many different groups have assessed these IgG4 deposits and uniformly they have confirmed their presence. In addition, studies on sIgG4 antibodies in serum of patients with EoE by Evan Dellon and Ben Wright at UNC in Chapel Hill, NC demonstrated sIgG4 antibodies to the major relevant proteins in EoE particularly to proteins from milk and wheat, in adults with EoE (36). At the same time, our group in collaboration with Dr. Elizabeth Erwin in Columbus, Ohio reported very high levels of sIgG4 to milk and wheat proteins in children with EoE (31, 37) (Figure 3). What matters here is that these findings provide support for a role for processing of milk and wheat in relation to the increasing prevalence of the disease. Indeed, we have recently published an article on the “processed milk hypothesis” which suggests that the droplets of milk fat in homogenized milk which have whey proteins as well as casein micelles bound to their surface are effectively “weaponized” to encourage an immune response in the esophageal wall (38). We also believe that these nanodroplets could act as a nidus for the IgG4 deposits in the esophageal wall biopsies. Notably the IgG4 antibodies in patients with EoE that are specific for alpha-lactalbumin (Bos d 4), beta-lactoglobulin (Bos d 5), and casein (Bos d 8) are at higher levels than any other specific antibodies of this isotype. In many cases, the levels of specific IgG4 antibodies to these proteins were as high or higher than 50 μg/mL. The level of detection (LOD) used in those assays for specific IgG4 was 100 ng/mL, however we used the number of sera at a level of ≥10 μg/mL for IgG4 to milk or wheat proteins to compare with the comparable number in a random control group in analyzing the risk for EoE (31) (Figure 2).

In 2023, an analysis of the nature of the IgG4 deposits in EoE was published by Emily McGowan and her colleagues at UVA that combined microscopy, coprecipitation and the STOMP technique to analyze the proteins present in these deposits (39). The results provide evidence that there are antigens related to cow's milk or wheat in the deposits, which strongly supports the view that these deposits consist of IgG4 antibodies specific for the main component antigens of cow's milk and wheat (37, 39). In turn the results could be explained if the deposits include nano-particles of cow's milk derived lipids coated with milk or wheat related allergens. As we have already mentioned, a controlled avoidance diet for EoE based on ISAC data was unsuccessful because ISAC cannot detect sIgE to milk proteins in the presence of high titer sIgG1 or IgG4 to the same milk proteins (Figure 2). Collectively the evidence strongly supports a role for processing of milk in relation to the increase in cases of EoE (38).

## 7 The identification of IgE antibodies specific for the oligosaccharide galactose alpha-1,3-galactose and their association with two novel forms of anaphylaxis

**Figure 4:**
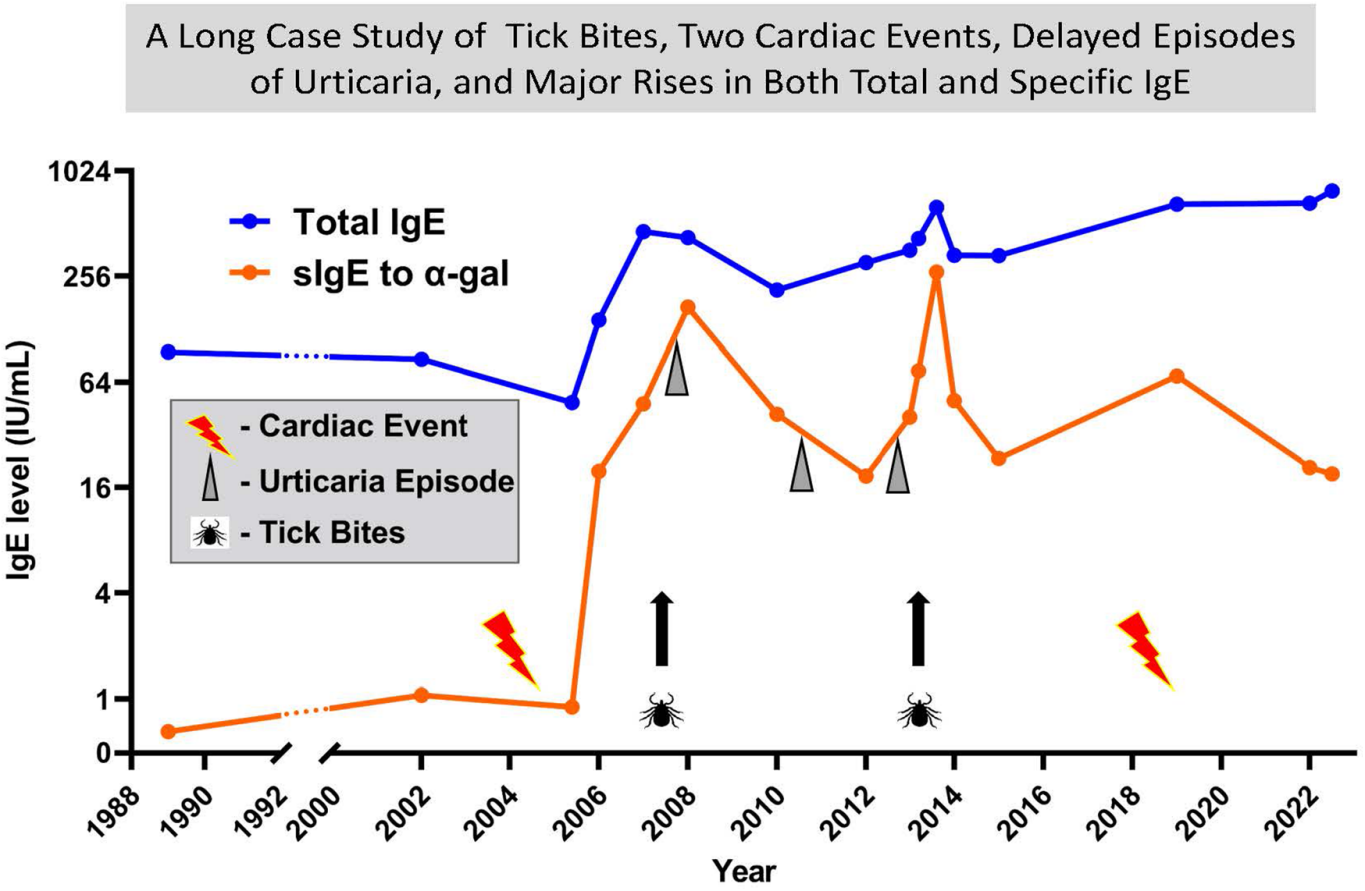
A thirty-four year case report with tick bites, two cardiac events, delayed episodes of urticaria and major rises in both total and specific IgE. A long-term follow up of total IgE and specific IgE to alpha gal on a patient who was 64 at the time of the first cardiac event. Significant urticaria events each started 4 or 5 hours after red meat. Tick bites have occurred at multiple times, but two significant events involved >10 bites. Since 2006 almost all tick bites have been prolonged pruritus.

The alpha-gal syndrome (AGS) induces both rapid, and often severe reactions to the first infusion of Cetuximab and also delayed but often severe reactions to meat and other products derived from non-primate mammals (27, 28, 40). In 2006, several patients and physicians had become aware of reactions to meat developing in adults who had experienced tick bites from one or more species of ticks (41). However, it was difficult to see how studying those cases was going to lead to the identification of the specificity of the immune response. Investigation by our group of the IgE antibodies in the serum of patients who had reacted to Cetuximab, in collaboration with the oncology group at Vanderbilt and Bristol, Myers, Squibb, collaboration with ImClone, which resolved the nature of the IgE epitope on Cetuximab (27). In particular, ImClone provided the molecule expressed in a cell line derived from Chinese hamster ovaries (CHO), to compare with the therapeutic molecule that was made in a mouse cell line SP2/O. Using streptavidin caps we were able to establish that the IgE antibodies in patients who reacted to their first infusion of Cetuximab only bound to the molecule expressed in SP2/O (27). That observation provided a clear indication that the target of the IgE antibodies was a post translational modification present on Cetuximab (27). In addition Dr. Zhou and his colleagues at ImClone provided the full data on glycosylation of Cetuximab which showed that the molecule carried galactose alpha-1,3-galactose on the Fab portion of the heavy chains (42). Following that it was relatively easy to prove that alpha-gal on the Fab portion of the heavy chain of Cetuximab was the target for the IgE antibodies (27). Within a year of our publication, Paul Parren and his colleagues in Amsterdam, with the help of some sera from patients in Virginia who had IgE antibodies to alpha-gal, had confirmed the evidence about Cetuximab as well as showing that alpha-gal on a molecule such as Infliximab which was on the Fc portion of the heavy chain was not accessible to serum IgE antibodies because it was encased within the two heavy chains (43). During the critical year when we proved the specificity of the IgE antibodies we had developed five separate assays for other molecules using the streptavidin ImmunoCAP and the “ImmunoCAP 250”. The realization that the Cetuximab CAP provided an excellent assay for IgE antibodies specific for alpha-gal allowed us to investigate hundreds of clinical cases. Those studies confirmed that IgE to alpha-gal was strongly associated with delayed anaphylaxis to meat or organs derived from non-primate mammals (28). This of course made sense since alpha-gal is the principle blood group substance of the non-primate mammals (44, 45). A patient who had been followed for various reasons for over 30 years developed Alpha-gal Syndrome (AGS) in his sixties having never had any symptoms of allergy to mammalian products before that. The episode started with greater than 200 bites from larval lone star ticks in August 2007, followed by a steady rise in sIgE to alpha-gal up to ∼90 IU/mL in November 2007. At that time, he had an extensive outbreak of hives that started 5 h after eating two lamb chops (Figure 4). Initially he had very low levels of sIgG4 to alpha-gal which is typical of new onset cases of AGS. However, more recently the levels of both sIgG4 and sIgG3 in sera from that patient have risen into the microgram range (Table 1). At present, we are not clear whether these antibodies would be protective or pro-inflammatory in the combination seen. Unfortunately, the FDA would not consider using an assay with Cetuximab on the solid phase as an assay for IgE antibodies to a widely distributed oligosaccharide. Because of that Phadia/Thermo Fisher were forced to develop an alternative assay, which resulted in ImmunoCAP using beef thyroglobulin (BTG) on the solid-phase (46). BTG is heavily decorated with alpha-gal in its natural form, and that assay was finally approved by the FDA in 2020. The discovery of the significance of IgE to alpha-gal incidentally led to a major increase of interest into the relevance of sIgE antibodies to oligosaccharides and the establishment of a working group by the IUIS/WHO subcommittee for allergen nomenclature in 2019. The report of that working group in 2022 was adopted as an addendum to the website in 2024 (www.allergen.org) (47).

**Table 1:** IgE, pan-IgG, IgG4, and IgG3 antibodies to galactose alpha-1,3-galactose.

Subject E036			Alpha-gal specific results*			
Event	Event date	Total IgE	IgE	pan IgG	IgG4	IgG3
		kU/L	kUA/L (%^#^)	ug/mL	ug/mL^+^	ug/mL
Blood draw	1989	94.9	0.04 0%	1.29	nd	0.29
Blood draw	April ‘02	49	0.51 1%	0.71	nd	<0.1
Cardiac event	March ‘05					
Blood draw	May ‘07	144	19.8 14%	3.05	nd	0.21
Sugar hollow ticks	Aug ‘07					
Blood draw	April ‘12	286	27.7 10%	1.58	0.09	<0.1
Pasture fence ticks	July ‘13					
Blood draw	Aug ‘13	334	40.5 12%	nd	nd	nd
Blood draw	Sept ‘13	633	89.3 14%	7.59	0.09	0.24
2nd cardiac event	Aug ‘18			3.55		
Blood draw	May ‘23	781	14.5 2%	5.88	0.33	1.2
Blood draw	Sept ‘23	1,350	116 9%	**19**.**6**	**4.76**	**9.2**

nd, not determined.

* Specific result assayed with beef thyroglobulin (BTG) ImmunoCAP 0.215.

^+^ IgG4 level of detection (LOD) ≥0.07 μL.

^#^ Specific IgE presented as % of total 1G4.

Bold values relate to values of sIgG4 or sIgG3 greater than or equal to 1 μ/mL.

## 8 Evidence relating to the cardiac risk associated with chronic exposure to food products derived from mammals or to a foreign body in the form of a bovine or porcine aortic valve

Antibody responses to the oligosaccharide galactose alpha-1,3-galactose have recently become an important area of investigation into the effects of chronic high level exposure to this epitope either as a glycoprotein or as a glycolipid (48, 49). Two different research groups have identified an association between increased severity of coronary artery disease (CAD) and the presence of IgE antibodies specific for alpha-gal (49, 50). Interestingly, in both of those studies, a large proportion of the subjects with specific IgE to alpha-gal were not aware of symptoms after eating red meat, and therefore had no reason to suspect this sensitivity.

While searching for further evidence about the association between coronary artery disease and specific IgE to alpha-gal, Jeff Wilson analyzed IgE antibodies in a cohort of subjects enrolled in the multiethnic study on atherosclerosis (MESA). In that cohort, which was enrolled at the Wake Medical Center, the prevalence of sIgE to alpha-gal was too low to see significant results, however there was a strong signal for the association between IgE to milk and cardiac mortality (51). That came to light from a collaboration between Corinne Keet of UNC and Jeff Wilson at UVA which found a significant relationship between sIgE to milk and cardiac disease in both NHANES and MESA (51). Given that there have been multiple studies in the past suggesting that a diet which includes mammalian products can increase the risk of cardiac disease the new data suggests that at least part of that risk relates to IgE antibodies specific for food antigens derived from mammals. On the other hand there has been some recent evidence from a cardiac surgery group in Vienna that the transplant of a biovalve derived from a pig or a cow can increase the production of sIgG3 antibodies to alpha-gal (52). Interestingly the isotype IgG3 has very little track record clinically. Until recently the evidence in relation to this association was based on ELISA assays. However, it is now possible to measure IgG3 antibodies with ImmunoCAP (53). The authors in Vienna clearly raised the question whether stimulation of sIgG3 antibodies to alpha-gal could have contributed to the increased mortality among patients who are relatively younger when they receive biovalve transplants. Currently mechanical valves, rather than biovalves, are recommended for patients under 55 years old, both in the USA and in Europe (54).

## 9 Relevance of isotype specific antibodies to component allergens from cat and mite that are germane to the effect of the indoor environment to asthma

In the early years of the 21st century, the average young person in western society spends as much as 90 to 95% of their time indoors. Although a large number of different allergen sources can be relevant to asthma, two sources stand out because we know a great deal about their component allergens and because they are relevant to a large number of individuals. The two allergen sources that we will focus on here are dust mites and cats. In particular, we are concerned with the details of the surprising phenomenon that some children who live in a home with a cat become immunologically tolerant to cat allergens (20, 55, 56). This phenomenon has also been studied in detail using both serum and cells from a small group of patients with asthma and an equal number of controls, who were living in homes with cats in Seattle (57). By contrast in almost all studies on the effects of dust mite exposure in houses, higher levels of exposure are associated with increased prevalence of sensitization (58, 59). Indeed the workshops on the risk of mite allergens to asthma concluded that there was a strong relationship between increased exposure to mite allergens and asthma (59). However, there is one study from Sydney, Australia that found a negative association between the highest levels of mite exposure and asthma (60). Furthermore, there has been some evidence that the mite component allergen with the highest exposure in houses may not always have the strongest association with asthma (22).

In 2001 we had data that some of the school children in the USA who lived in a home with a cat and had correspondingly high exposure to the cat allergen Fel d 1 had high levels of IgG and specifically IgG4 antibodies to Fel d 1 (20). More important many of those individuals did not have asthma and were clinically tolerant to exposure to cat allergens (56). We found similar data in cohorts of school children living in an area of Sweden where because of the climate there was no exposure to dust mites, cockroaches, or mold antigens (61). Perhaps more striking we found that children in New Zealand who lived in houses with high exposure to dust mite and cat allergens were less likely to be sensitized to cat allergens but that the presence of a cat in the home had no effect on sensitization to mite allergens (62). Thus, among 50 children with asthma in the New Zealand study who were living in a house with a cat, 34 were sensitized to mite allergens but not sensitized to cat allergens (62). Most of the studies on IgG4 antibodies carried out before 2005 were carried out with ELISA or a double antibody precipitation assay which was technically very difficult. However, in 2010 the ImmunoCAP assay was adapted by Phadia to assay specific IgG4 antibodies using the same allergen caps that are used for the IgE assays: This is the same IgG4 assay that we used for measuring specific IgG4 antibodies to milk and wheat proteins in sera from patients with Eosinophilic Esophagitis (31, 37). We now have evidence that those component allergens of both cat and mite with the highest abundance in houses produce high levels of IgG4 and that this can be associated with a less convincing relationship to asthma (22). By contrast, the specific allergens that have lower abundance stimulate lesser quantities of IgG4 antibodies and can have a stronger relationship to asthma (22).

## 10 Conclusions

Over the last 50 years there have been major advances in our ability to measure allergen specific antibodies of several different isotypes. In large part, this has focused on the identification and accurate measurement of IgE as a separate isotype which is uniquely able to bind to the Fc Epsilon Receptor1 (FcεR1) and to activate both mast cells and basophils. However, there are two other areas of research that have changed this area of study:

(i) The first is a progressive ability to identify and purify specific allergens both by immunochemical techniques and more recently by cloning (see www.allergen.org).
(ii) The second was the development of monoclonal antibodies that made it possible to accurately identify and measure allergen specific antibodies of both IgE and other isotypes.

Interestingly when Clemens Von Pirquet introduced the word “allergy” in 1906 he said that the word should apply to all substances that could induce an allergic response, but he added that the word should also apply to “those substances that give rise to super sensitivity without immunity” (63, 64). Over time this final phrase has become in effect the definition of the word allergen, however many people have argued about what Von Pirquet meant by the words “without immunity”. In 1921 when Prausnitz and Kustner published their primary paper on P-K reactions they mentioned that the two forms of immunity that Von Pirquet had referred to were precipitation reactions with an allergen in a gel and complement fixation (2). Interestingly, this focuses on two aspects of the immune response that are features of different isotypes, certainly IgE antibodies are not known to give rise to precipitation or to activate complement (5). Equally allergen specific antibodies of the IgG4 isotype do not give precipitation reactions or activate complement (65). As we have discussed here, there are multiple areas of allergic disease where IgG4 antibodies have a role:

The first is that the response to repeated exposure to inhalant allergens, such as (i) many years of pollen exposure, (ii) the response to immunotherapy (iii) high exposure to a cat at home or (iv) exposure of employees to small animal allergens in a vivarium (66). The second is the IgG4 antibody response to protein allergens from cow's milk or wheat; where both the exposure to these proteins and the levels of sIgG4 antibodies in the serum of patients with EoE are 10 to 100 fold higher than the levels seen with inhalant allergens (37) (Figure 3). Thus there are now several clinical situations where the ability to measure sIgG4 antibodies is an important part of research and may also be helpful in clinical practice.

Prior to the current technical revolution, Robert Cooke in New York demonstrated that there were at least two different aspects of the immune response to pollen allergens: those antibodies (generally called reagins) that could transfer skin test reactivity (P-K activity) and also antibodies that could block skin reactivity (blocking antibodies) (67). Blocking antibodies undoubtedly include both IgG1 and IgG4 antibodies. However, the antibody response to immunotherapy may also include IgA antibodies (14). Standard assays for sIgG1 antibodies have problems because the total quantities of IgG1 in the circulation range from 5 to 12 mg/mL which means that with almost any form of assay the background binding of IgG molecules to the relevant allergen is very high. Although IgG1 antibodies can be measured with either ELISA or precipitation assays, the serum generally has to be diluted by 25 fold or 100 fold before making any measurements. With the earlier assays the results for IgG4 or other IgG isotypes were generally given in arbitrary or ELISA units. If the values were calculated further, the lower level of detection was generally as high as 1μg/mL. With the newer assays values for specific IgG4 can be given at 100 ng/mL or lower. Clearly, it is important for authors, reviewers, and editors to make all efforts to keep the units correct.

Over the last 50 years, there have been progressive improvements in the techniques to measure allergen specific antibodies of different isotypes. The most obvious of these relate to simplicity and accuracy of specific IgE antibodies; which are now relevant to the diagnosis and management of a wide range of allergic diseases. In addition, techniques for measuring other isotypes such as pan-IgG, IgG4, and IgG3 are already playing significant roles in understanding; the response to immunotherapy, Eosinophilic esophagitis, and the possible chronic effects of exposure to the oligosaccharide alpha-gal in the diet of patients with specific IgE antibodies. In short techniques for the measurement of allergen specific antibodies of multiple isotypes are going to be available and will be essential for the continued investigation of allergic disease.
